# Supplementary material for: LncBRM initiates YAP1 signalling activation to drive self-renewal of liver cancer stem cells
Source: Nat Commun. 2016 Dec 1;7:13608. doi: 10.1038/ncomms13608 (PMC5146280; doi:10.1038/ncomms13608)
Supplement: Supplementary Information — Supplementary Figures 1-8 and Supplementary Tables 1-4. [file ncomms13608-s1.pdf]

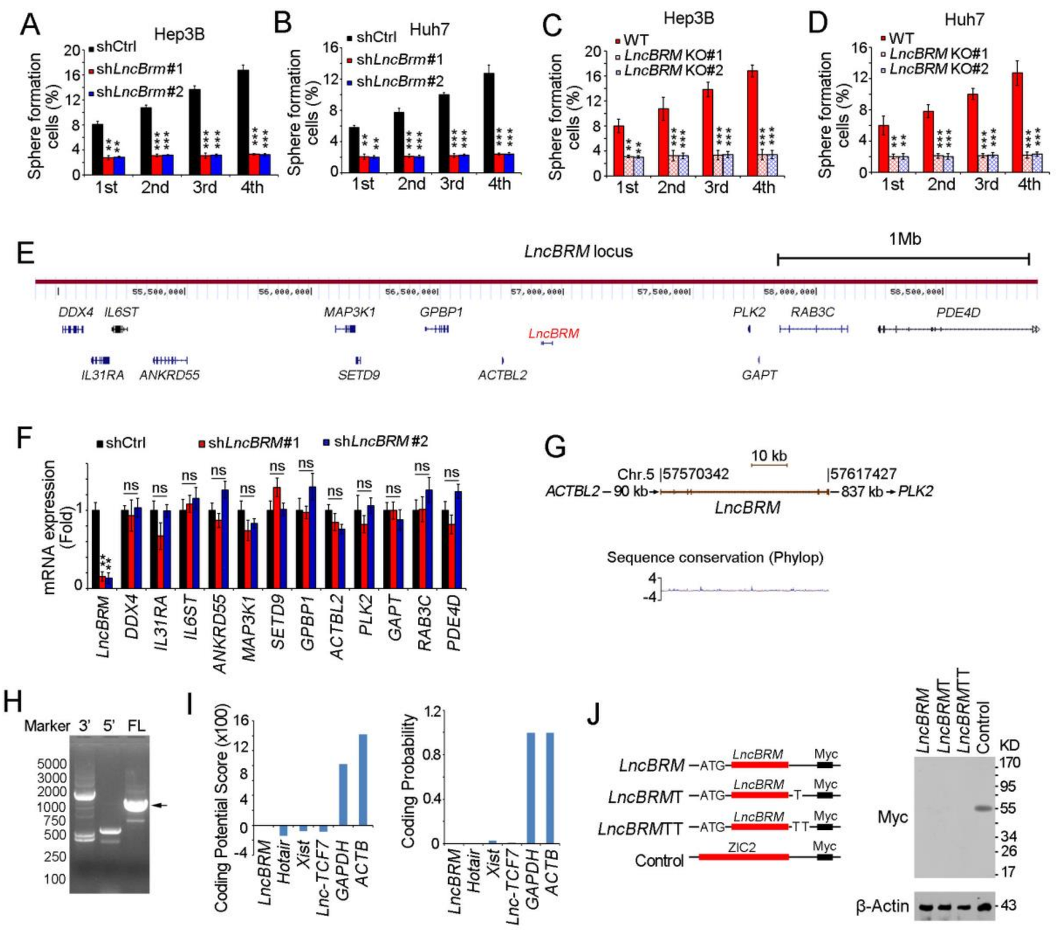

Supplementary Figure 1. *Lnc-β-Catm* characterization. (A, B) *LncBRM* silenced Hep3B (A) and Huh7 (B) cells were established using pSiCoR lentivirus, followed by sphere formation assays. (C, D) LncBRM deleted Hep3B (C) and Huh7 (D) cells were established using a CRISPR/Cas9 approach, followed by serial sphere formation assays. (E) 12 genes nearby *lncBRM* locus (less than 2 Mb) were shown. Location information was obtained from UCSC dataset (http://genome.ucsc.edu/cgi-bin/hgGateway). (F) *LncBRM* silenced and shCtrl spheres were collected, followed by examination of gene expression levels by realtime PCR. (G) Schematic annotation of *lncBRM* genomic locus on chromasome 5. Purple rectangles represent exons (upper panel), sequence conservation was analyzed by Phylop software (lower panel). (H) Full length of *lncBRM* was examined by 3' and 5' rapid-amplification of cDNA ends (RACE) assays. The length of *lncBRM* was 1321 nucleotides. Black arrowhead indicates the full length of *lncBRM*. (I) Coding potential of *lncBRM* was analyzed using CPC (left panel) and CPAT (right panel). HOX transcript antisense RNA (*Hotair*), X inactivation-specific transcript (*XIST*) and

lnc-TCF7 served as control non-coding RNAs. β-actin (*ACTB*) and Glyceraldehyde-3-phosphate dehydrogenase (*GAPDH*) served as control coding genes. CPC scores represent the coding ability; CPAT scores indicate the possibility of coding. (J) The indicated genes were cloned into PCDNA4-HisMycB vector and transfected into Huh7 cells. 2 days later, cells were lyzed with RIPA buffer, followed by Western blot with Myc antibody. ZIC2 served as a coding control. Data are shown as means ± SD. Two tailed Student's t-test was used for statistical analysis, **, $p < 0.01$; ***, $p<0.001$; ns, not significant. Data are representative of three independent experiments.

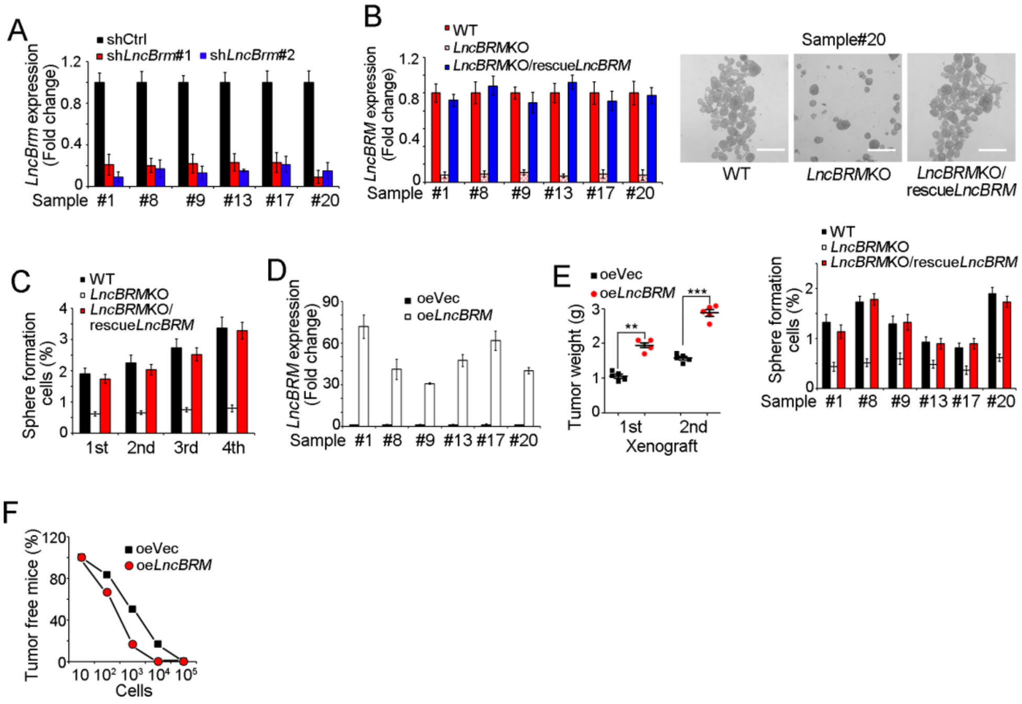

Supplementary Figure 2. *LncBRM* promotes self-renewal of liver CSCs. (A) *LncBrm* silenced primary cells were established using pSiCoR lentivirus and confirmed by realtime PCR. shCtrl, Control shRNA. (B) *LncBRM* knockout cells were established using a CRISPR/Cas9 approach (left panel), followed by sphere formation. Representative sphere images were shown in the right panel and statistic ratios were shown in the lower right panel. (C) Self-renewal of *lncBRM* knockout and rescued HCC cells were detected by serial sphere formation assays. (D) *LncBRM* overexpressing primary cells were established using lentivirus, and the overexpressing efficiency was examined by realtime PCR. oeVec, overexpressing Vector control; oe*LncBRM*, overexpressing *lncBRM.* (E) *LncBRM* overexpressing and control primary cells were established with pSiCoR lentivirus and used for subcutaneous injection. Xenograt tumors were used for serial tumor implantation. (F) LncBrm overexpressing and control cells (10, 10^2, 10^3, 10^4 and 10^5) were subcutaneously injected into BALB/c nude mice. Tumor-free mice ratios are calculated three month later. n=6 for each group. Data are shown as means ± SD. Two tailed Student's t-test was used for statistical analysis, *, $p < 0.05$; **, $p < 0.01$; ***, $p < 0.001$. Data are representative of four independent experiments.

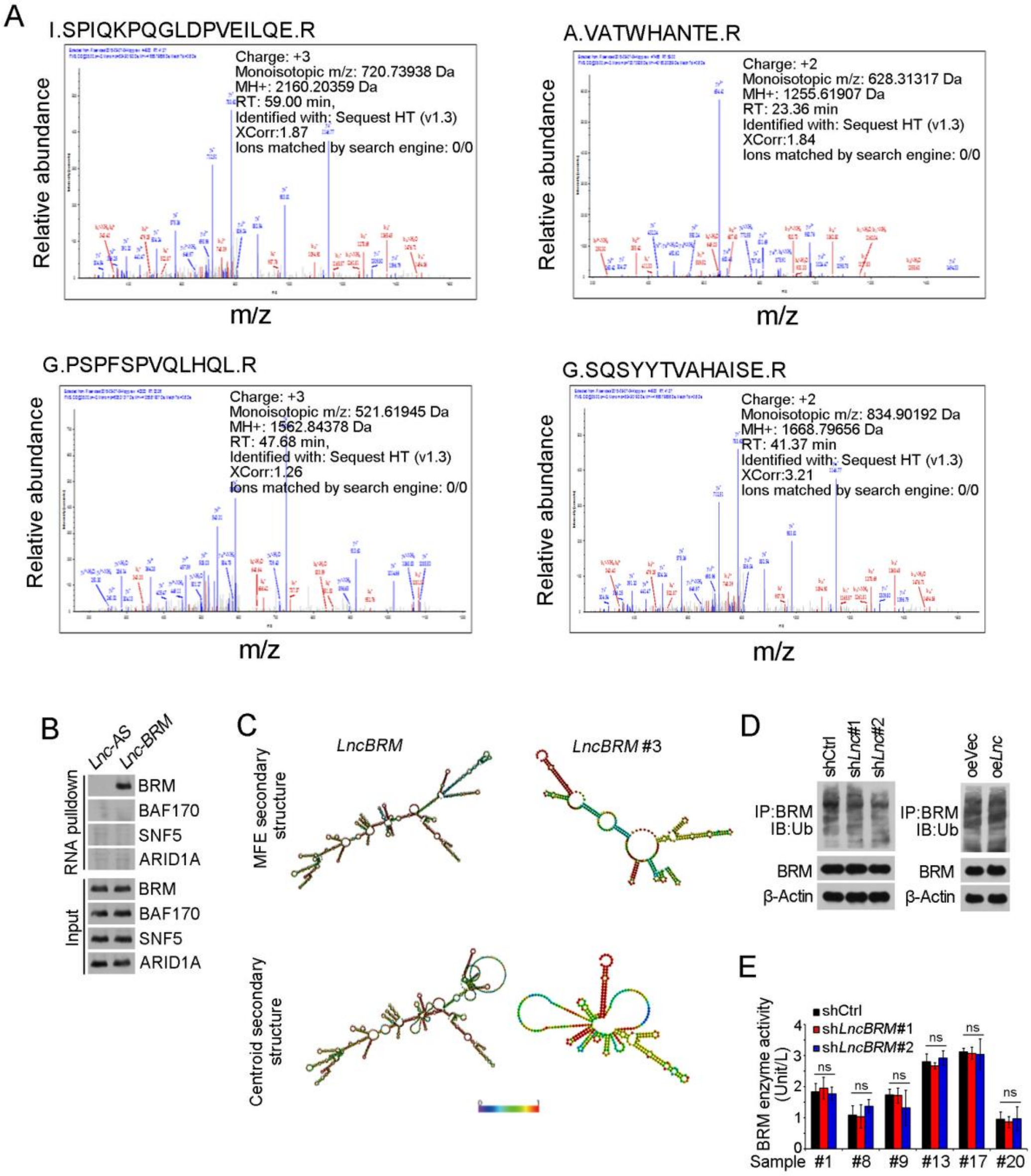

Supplementary Figure 3. *LncBRM* interacts with BRM. (A) MS/MS profiles of BRM. Corresponding peptide sequences are listed on top of the corresponding graphs. (B) *LncBRM* interacts with free BRM but not other BAF components. *LncBRM* was labeled with biotin using in vitro transcription and incubated with oncosphere lysates for RNA pulldown assay. Precipitates were subjected to SDS-PAGE for separation, and detected by Western blot with the indicated antibodies. (C) Full length (1-1321 nt) and segment #3 (607-951 nt) of *lncBRM* were predicted to harbor stable stem-loop structures. Predictions were based on minimum free energy (MFE) and partition function. Color scales denote confidence of predictions for each base with shades of red indicating strong confidence (http://rna.tbi.univie.ac.at/). (D) *LncBRM* has no impact on BRM stability. *LncBRM*

silenced and shCtrl spheres were lyzed for immunoprecipitation with BRM antibody, followed by immunoblotting using anti-ubiquitin (Ub) antibody. (E) *LncBRM* has no effect on BRM ATPase activity. Precipitated BRM from *lncBRM* silenced or shCtrl spheres was measured for ATPase activity by ATPase/GTPase Activity Assay Kit (Sigma). Data are shown as means ± SD. ns, not significant. Data are representative of three independent experiments.

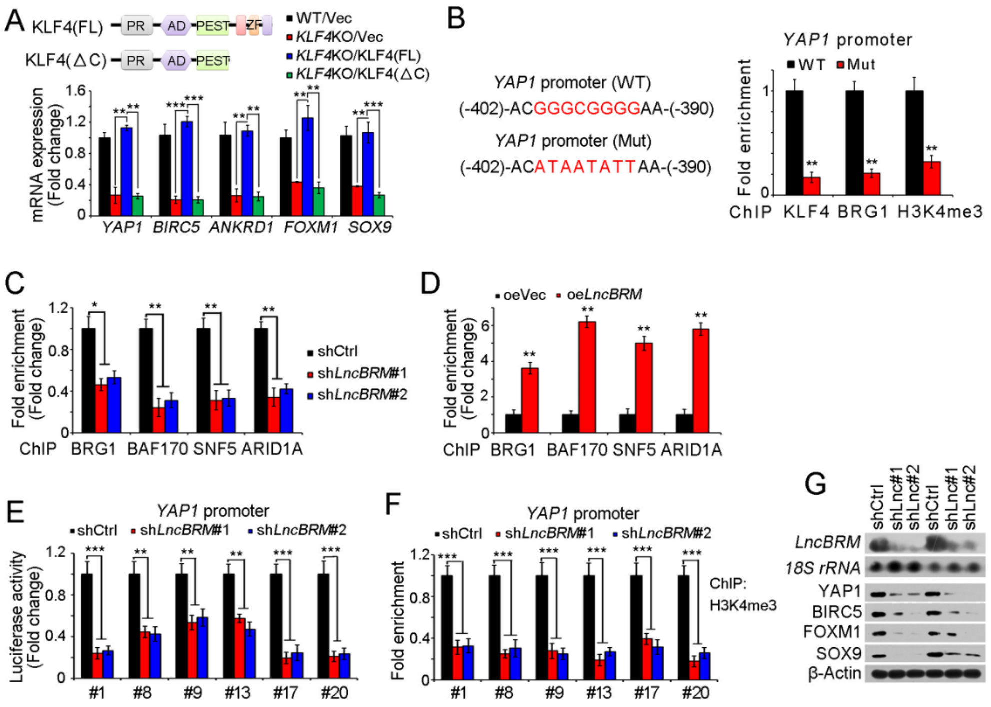

Supplementary Figure 4. KLF4 plays a critical role in activation of YAP1 signaling. (A) Full length (FL) and C terminal truncate (△C) KLF4 were cloned into pBPLV vector and transfected into *KLF4* knockout cells, followed by sphere formation assay. YAP1 signaling target genes were examined by realtime PCR. PR, proline-rich domain; AD, acidic domain; PEST, PEST domain, ZF, Zinc finger domain. (B) KLF4 binding sequence (GGGCGGGG) was replaced with mutant sequence (ATAATATT) (left panels) by a CRISPR/Cas9 approach. WT and mutant Huh7 cells (Mut) were collected for ChIP assays with KLF4, BRG1 and H3K4me3 antibodies, and *YAP1* promoter enrichment was examined using realtime PCR. (C, D) BRG1, BAF170, SNF5 and ARID1A ChIP assays were performed using *lncBRM* silenced (C) and overexpressing (D) cells. (E, F) Activation of *YAP1* promoter was examined by luciferase assay (E) and H3K4me3 ChIP assay (F) in *lncBRM* silenced spheres. (G) *LncBRM* silenced cells were established (upper panels), followed by Western blot (lower panels). 18S rRNA served as a loading control for Northern blot, and β-actin served as a loading control for Western blot. **, $p < 0.01$; ***, $p < 0.001$. Data are representative of three independent experiments.

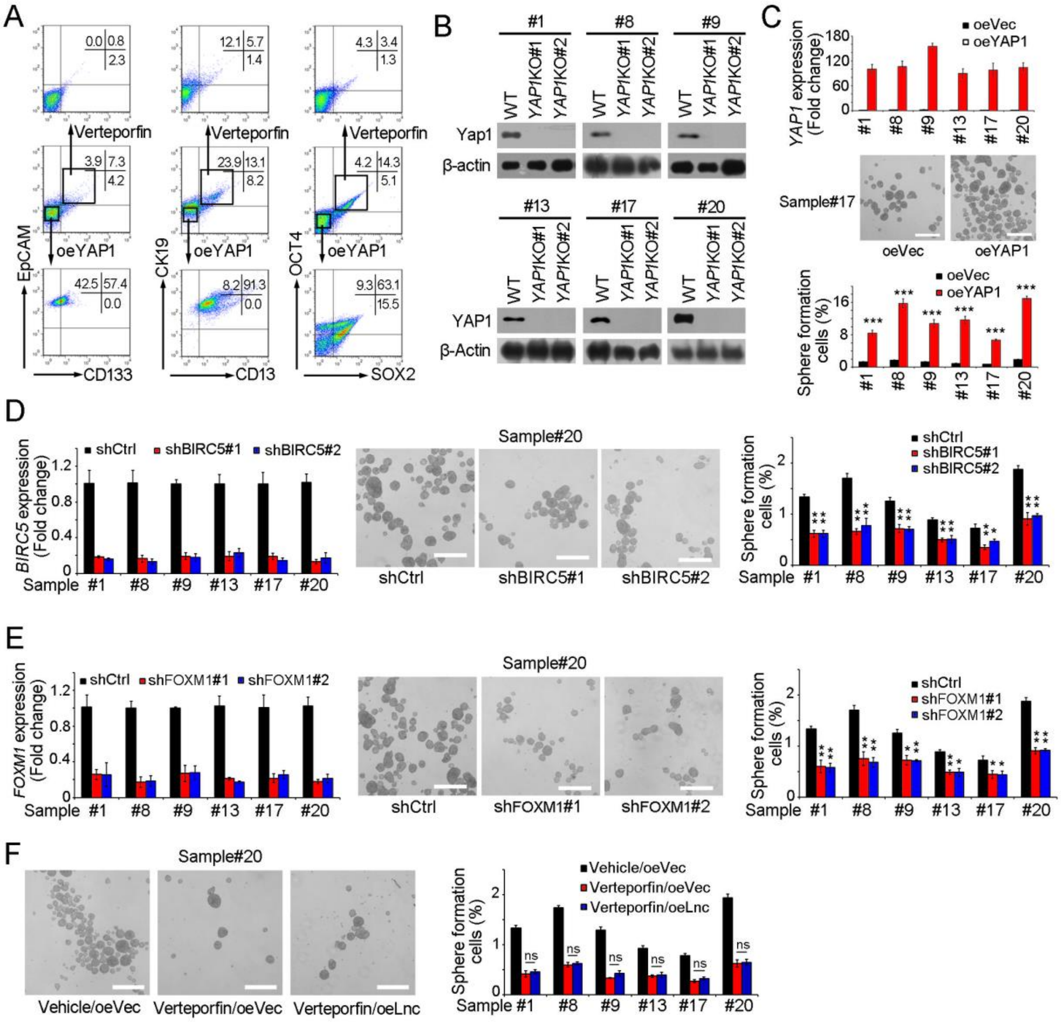

Supplementary Figure 5. YAP1 signaling is required for self-renewal of liver CSCs. (A) Liver CSCs and non-CSCs were treated with Verteportin and YAP1 overexpression, respectively. Liver CSC self-renewal was detected using the indicated CSC markers and stem factors. (B) YAP1 deficient cells were established using a CRISPR/Cas9 approach, and validated with Western blot. WT, wild type; *YAP1*KO, *YAP1* knockout. (C) YAP1 overexpressing cells were established and followed by sphere formation assays. (D, E) Birc5 or Foxm1 silenced cells were established (left panels), followed by sphere formation assays. (F) *LncBRM* overexpressing cells were treated with YAP1 inhibitor Verteporfin, followed by sphere formation assays. oeVec, overexpression vector; oeLnc, overexpression *lncBRM*. For C, D, E, F, scale bars, 500 μm. Data are shown as means ± SD. *, $p < 0.05$; ***, $p < 0.001$. ns, not significant. Data are representative of four independent experiments.

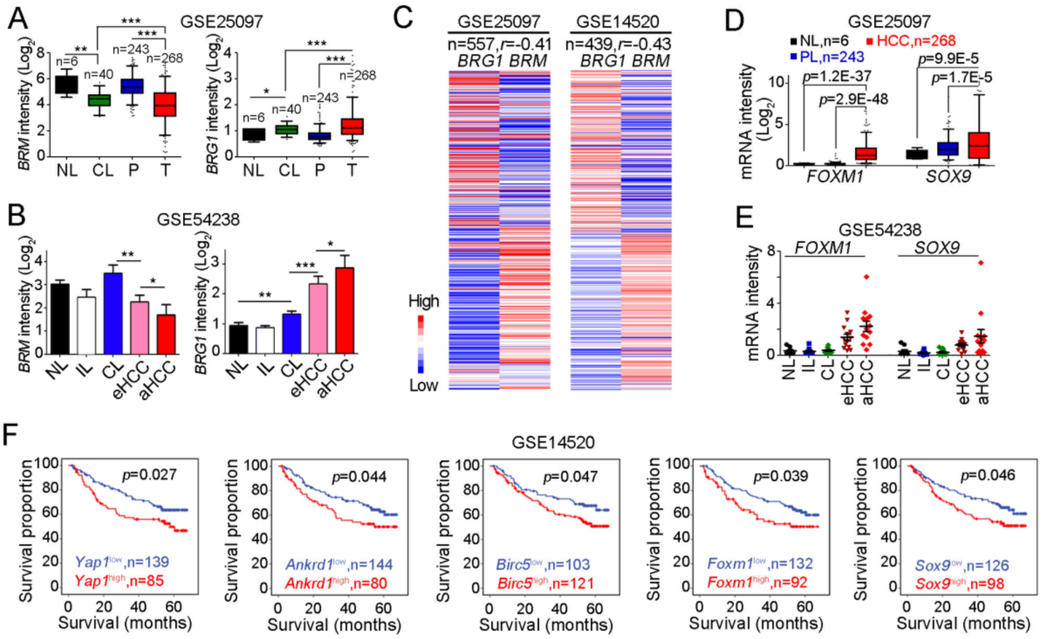

Supplementary Figure 6. Expression levels of BRG1 and YAP1 targets are positively related to cancer severity of HCC. (A, B) Decreased *BRM* expression (left panels) and increased *BRG1* expression (right panels) in HCC tumor tissues derived from Zhang's cohort (GSE25097, A) and Wang's cohort (GSE54238, B). R language was used for gene expression analysis. P, peri-tumor; T, tumor; NL, normal liver; CL, cirrhosis liver; eHCC, early HCC; aHCC, advanced HCC. (C) Expression levels of *BRG1* and *BRM* were analyzed using Zhang's cohort (GSE25097) and Wang's cohort (GSE14520) datasets using R language, and shown as heat map. r, Pearson correlation coefficient. (D) Higher expression levels of YAP1 target genes in HCC tumors were validated using Zhang's cohort (GSE25097). There were no probes for *Lats1*, *YAP1* and other YAP1 target genes. NL, normal liver; PL, peri-tumor liver tissues. (E) Elevated *Foxm1* and *Sox9* expression appeared in advanced HCC patients derived from Wang's cohort. NL, normal liver; IL, inflammatory liver; CL, cirrhosis liver; eHCC, early HCC; aHCC, advanced HCC. (F) Expression levels of *YAP1* and its target genes were related to clinical prognosis of HCC patients. HCC samples were divided into two groups according to the indicated gene expression levels, followed by Kaplan–Meier survival analysis. For A, D: data are shown as box and whisker plot. Box: interquartile range (IQR); horizontal line within box: median;

whiskers: 5–95 percentile. For B, E, data are shown as means ± SD. Two tailed Student's t-test was used for statistical analysis.

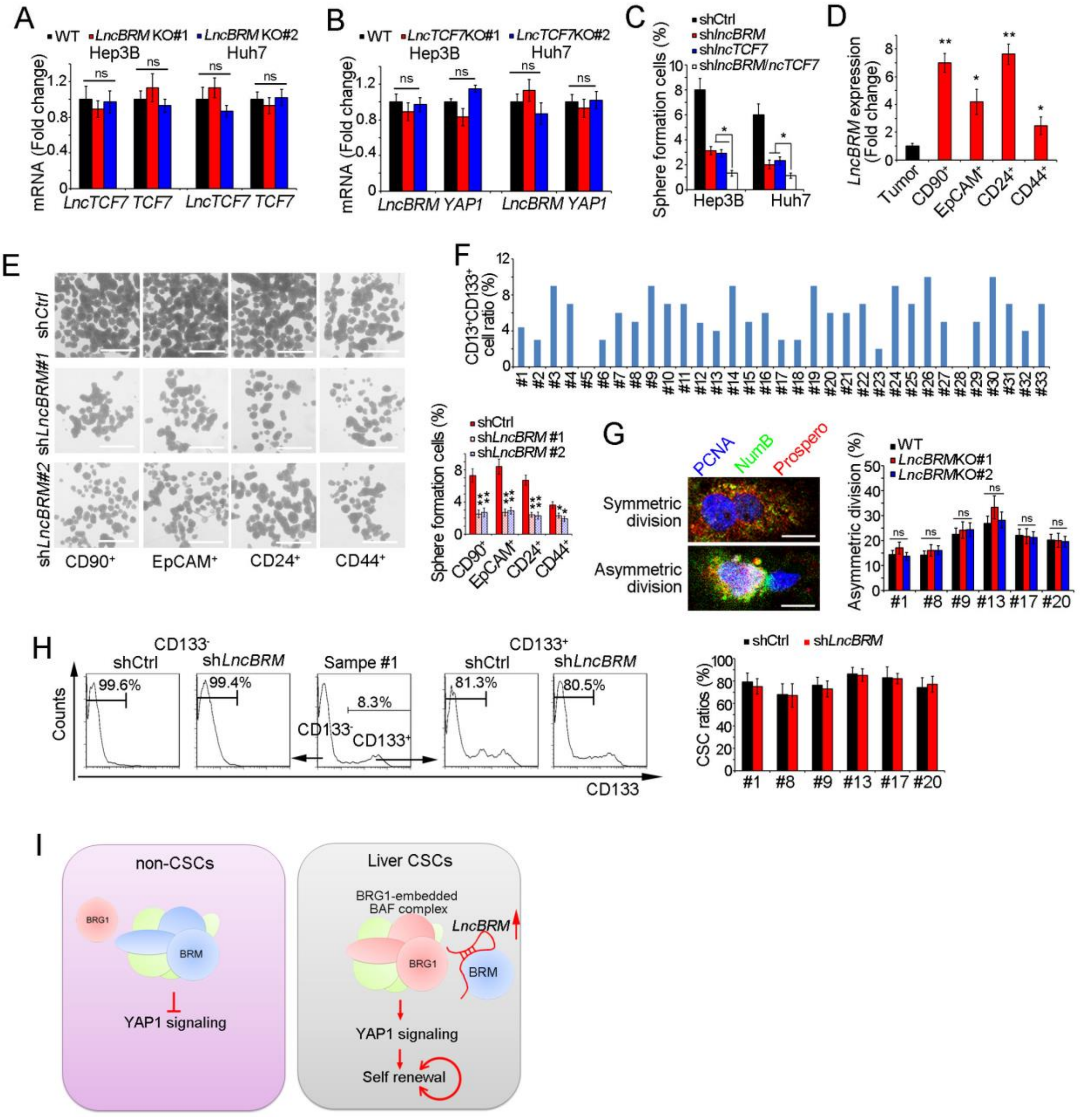

Supplementary Figure 7. *LncBRM* drives YAP1 activation in liver CSCs. (A, B) *LncBRM* and *lncTCF7* knockout cells were established using a CRISPR/Cas9 approach, followed by mRNA detection by realtime PCR. (C) *LncBRM* silenced, *lncTCF7* silenced or double knockdown cells were established using PSiCoR lentivirus, followed by sphere formation assays. (D) $CD90^+$, $EpCAM^+$, $CD24^+$ or $CD44^+$ Cells were enriched by FACS, followed by realtime PCR for *lncBRM* expression. (E) $CD90^+$, $EPCAM^+$, $CD24^+$ or $CD44^+$ cells were isolated using FACS and infected with shlncBRM virus or shCtrl control virus, followed by sphere formation assays. Representative images were shown in the left panel and sphere ratios shown in the right panel. (F) $CD13^+CD133^+$ cells were detected using FACS, and ratios were shown as histogram. (G) $CD13^+CD133^+$ cells were enriched and seeded onto cover slips for 2 days in incubator, and then cells were fixed, penetrated and

stained with the indicated antibodies. Representative images were shown in the middle panel and statistic ratios were shown in the right panel. Asymmetric division (%) = asymmetric division / (asymmetric division + symmetric division). (H) $CD133^+$ and $CD133^-$ cells were sorted with FACS, followed by lncBRM knockdown and subsequent long-term culture for differentiation assays. Ratios of $CD133^-$ cell differentiation were analyzed by FACS. Statistic ratios of $CD133^-$ cells on day 20 were shown in the right panel. (I) In non-CSCs, *lncBRM* is much lowly expressed, the BRM-embedded BAF complex exists, which blocks YAP1 signaling activation. By contrast, in liver CSCs, *lncBRM* is much highly expressed, *lncBRM* interacts with BRM to sequester BRM away from the BAF complex, facilitating the formation of BRG1-biased BAF complex, leading to YAP1 signaling activation. Consequently, *lncBRM*-mediated YAP1 signaling activation regulates the self-renewal of liver CSCs and their tumorigenesis. Scale bars, E, 500 μm; G, 10 μm. Data are shown as means ± SD. Two tailed Student's t-test was used for statistical analysis, *, $p < 0.05$; **, $p < 0.01$; ns, not significant. Data are representative of three independent experiments.

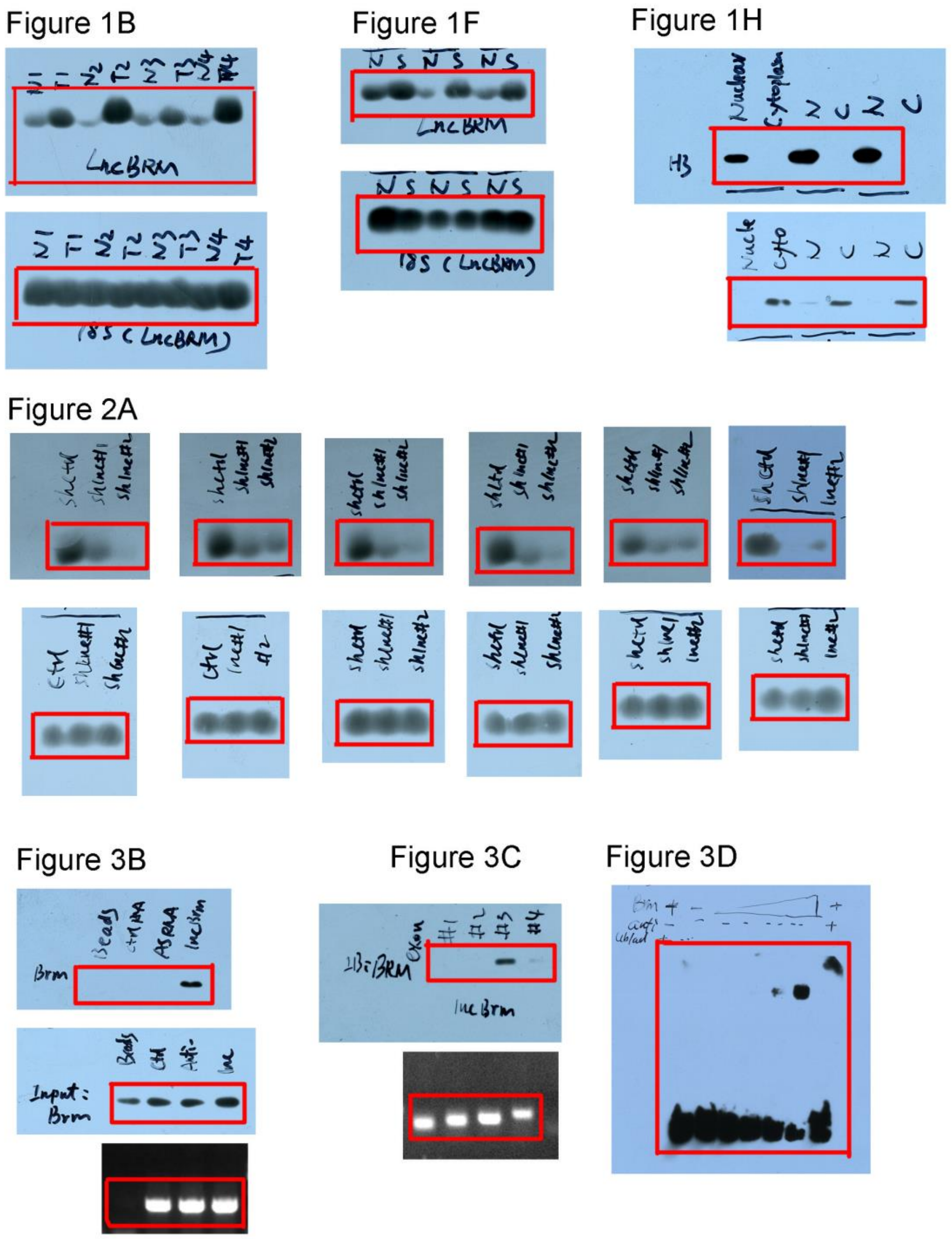

Supplementary Figure 8. Uncropped blots of figures. The red sections indicate blot results shown in the indicated figures.

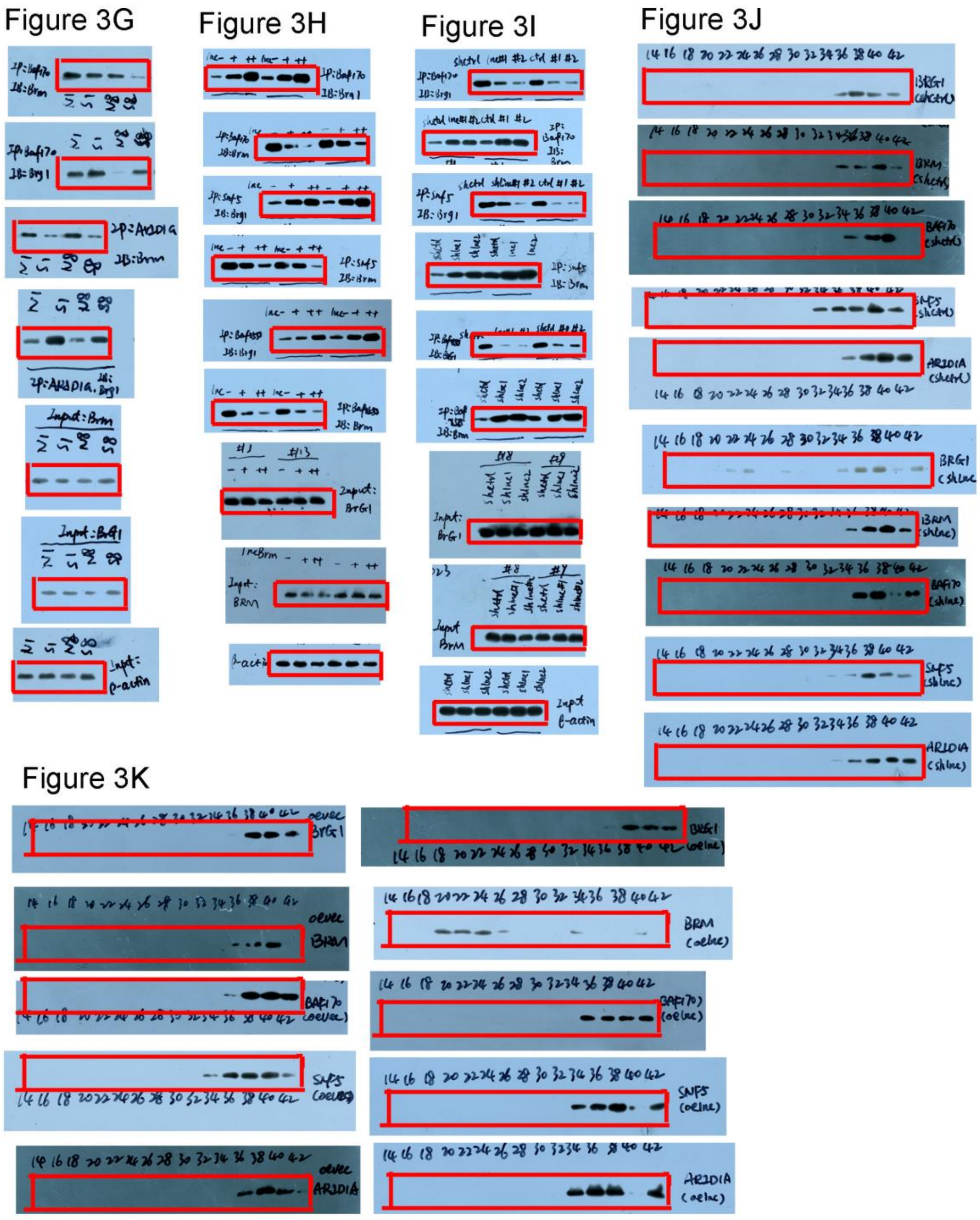

Supplementary Figure 8. Uncropped blots of figures. The red sections indicate blot results shown in the indicated figures.

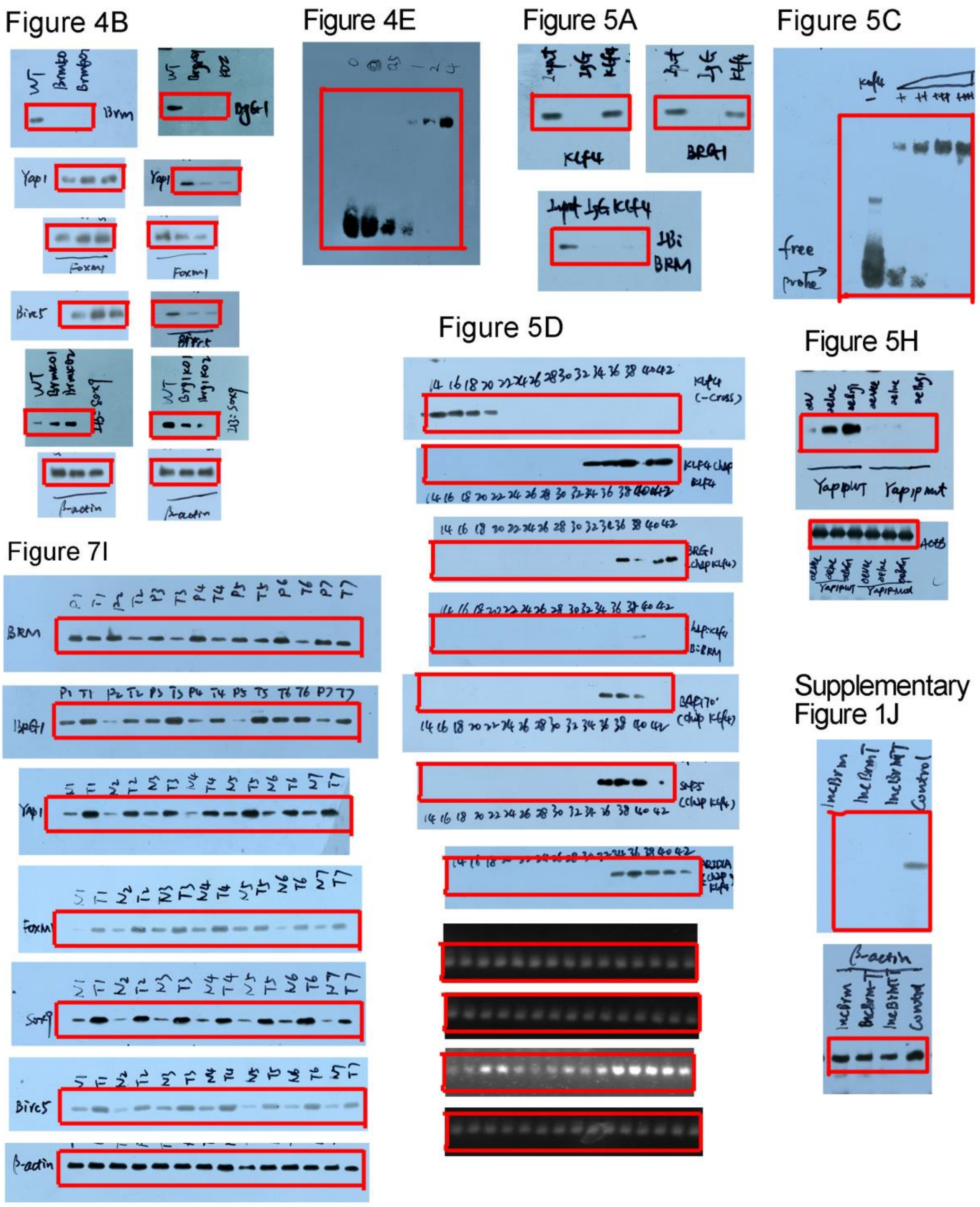

Supplementary Figure 8. Uncropped blots of figures. The red sections indicate blot results shown in the indicated figures.

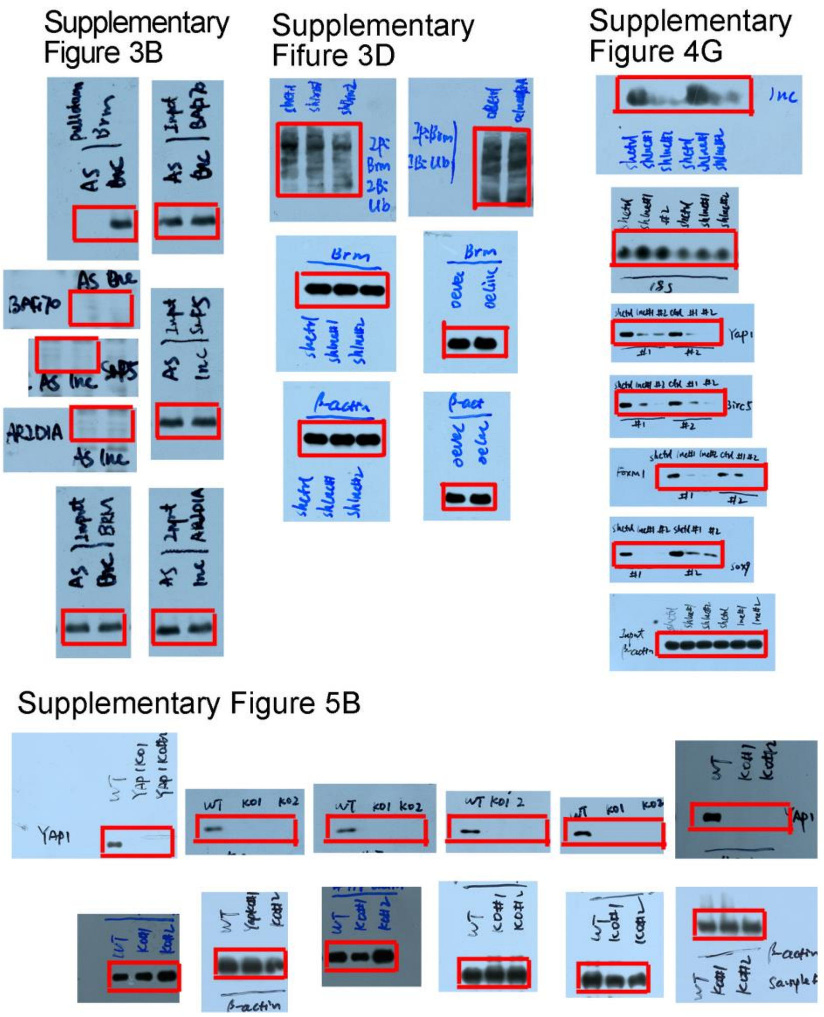

Supplementary Figure 8. Uncropped blots of figures. The red sections indicate blot results shown in the indicated figures.

Supplementary Table 1. Clinical information of HCC patients we studied

Sample	Diagnosis	Differentiation	Metastasis	Size (cm)	Stage	Tumor location
1#	HCC	low	no	9×7×6.5	III	left lobe
8#	HCC	low	no	2.5×2.5×2.5	III	right anterior lobe
9#	HCC	medium	Yes	7×6×6	II	Left lateral lobe
13#	HCC	low	Yes	7×6×6	III	left lobe
17#	HCC	medium	yes	10×8.5×6	II	right lobe
20#	HCC	low	yes	6.5×3.5×3	II-III	right lobe

Supplementary Table 2. CSC ratios of the indicated cells.

A

Cell	CSC ratio (95% CI)	*P* value
shCtrl (A)	1/3190 (1/8046-1/1264)	
shLncBRM #1 (B)	1/12241 (1/33426-1/4483)	0.029 (B vs A)
shLncBRM #2 (C)	1/27311 (1/71229-1/10472)	0.0002 (C vs A)

B

Cell	CSC ratio (95% CI)	*P* value
oeVec (A)	1/3190 (1/8046-1/1264)	
oe*LncBRM* (B)	1/459 (1/1109-1/190)	0.002 (A vs B)

C

Cell	CSC ratio (95% CI)	*P* value
WT (A)	1/3190 (1/8046-1/1264)	
*YAP1*KO#1 (B)	1/26597 (1/69798-1/10135)	0.0002 (B vs A)
*YAP1*KO#2 (C)	1/12241 (1/33426-1/4483)	0.029 (C vs A)

10, 1×10^2, 1×10^3, 1×10^4 and 1×10^5 indicated cells were subcutaneously injected into BALB/c nude mice on the back and tumor formation was observed three months later. Liver CSC ratios were calculated using extreme limiting dilution analysis. CI, Confidence interval; vs, versus.

Supplementary Table 3. Realtime PCR primers used in this study

Primers	Sequences
18S (Forward)	5’-AACCCGTTGAACCCCATT-3’
18S (Reverse)	5’-CCATCCAATCGGTAGTAGCG-3’
actin (Forward)	5’-TCCATCATGAAGTGTGACGT-3’
actin (Reverse)	5’-GAGCAATGATCTTGATCTTCAT-3’
LncBRM (Forward)	5’-GGTCAAGAGGCCAGGAAGAG-3’
LncBRM (Reverse)	5’-TTCTCACTTCAGCCCAATGCT-3’
BRG1 (Forward)	5’-CAGATCCGTCACAGGCAAAAT-3’
BRG1 (Reverse)	5’-TCTCGATCCGCTCGTTCTCTT-3’
BRM (Forward)	5’-AGGGGATTGTAGAAGACATCCA-3’
BRM (Reverse)	5’-TTGGCTGTGTTGATCCATTGG-3’
Ctgf (Forward)	5’-CAGCATGGACGTTCGTCTG-3’
Ctgf (Reverse)	5’-AACCACGGTTTGGTCCTTGG-3’
YAP1 (Forward)	5’-TAGCCCTGCGTAGCCAGTTA -3’
YAP1 (Reverse)	5’-TCATGCTTAGTCCACTGTCTGT-3’
Ankrd1 (Forward)	5’-AGTAGAGGAACTGGTCACTGG -3’
Ankrd1 (Reverse)	5’-TGTTTCTCGCTTTTCCACTGTT -3’
Birc5 (Forward)	5’-CCAGATGACGACCCCATAGAG -3’
Birc5 (Reverse)	5’-TTGTTGGTTTCCTTTGCAATTTT-3’
Foxm1 (Forward)	5’-CGTCGGCCACTGATTCTCAAA-3’
Foxm1 (Reverse)	5’-GGCAGGGGATCTCTTAGGTTC-3’
Sox9 (Forward)	5’-AGCGAACGCACATCAAGAC-3’
Sox9 (Reverse)	5’-CTGTAGGCGATCTGTTGGGG-3’
DDX4 (Forward)	5’-TCATACTTGCAGGACGAGATTTG-3’
DDX4 (Reverse)	5’- AACGACTGGCAGTTATTCCATC-3’
IL31RA (Forward)	5’-CACACTTCGATTCAGGACAGTC-3’
IL31RA (Reverse)	5’-CACATCGCAGAGCTATGACAT-3’
IL6ST (Forward)	5’-GTGAGTGGGATGGTGGAAGG-3’
IL6ST (Reverse)	5’-CAAACTTGTGTGTTGCCCATTC-3’
MAP3K1 (Forward)	5’-CATCAGGTCGCACAGTGAAAT-3’
MAP3K1 (Reverse)	5’-TCAGGGCTATATGGTGAGAAGC-3’
SETD9 (Forward)	5’-GCCGTTACAAGTACCGCTTC-3’
SETD9 (Reverse)	5’-CTCTGGAACATATCGGAGGGT-3’
GPBP1 (Forward)	5’-GCAGCCTCGTCTAACCAAACT-3’
GPBP1 (Reverse)	5’-CAGCACGGCTTTCATCTTCAT-3’
ACTBL2 (Forward)	5’-CTCGACACCAGGGCGTTATG-3’
ACTBL2 (Reverse)	5’-CCACTCCATGCTCGATAGGAT-3’
PLK2 (Forward)	5’-CTACGCCGCAAAAATTATTCCTC-3’
PLK2 (Reverse)	5’-TCTTTGTCCTCGAAGTAGTGGT-3’
GAPT (Forward)	5’-AAGCAGGTCCTCCCAAAGCTA-3’
GAPT (Reverse)	5’-CCTCGAAATTAGACTGCCCTGT-3’
RAB3C (Forward)	5’-GGAAGACGAGCGGGTCATC-3’
RAB3C (Reverse)	5’-CTCTCTGACATTTTGTCGCAGAT-3’
PDE4D (Forward)	5’-ACGGACCGGATAATGGAGGAG-3’
PDE4D (Reverse)	5’-ATTTTTCCACGGAAGCATTGTG-3’
VEGF (Forward)	5’-ATCACGAAGTGGTGAAGTTC-3’
VEGF (Reverse)	5’-TGCTGTAGGAAGCTCATCTC-3’
Bcl2l1 (Forward)	5’-GAGCTGGTGGTTGACTTTCTC-3’
Bcl2l1 (Reverse)	5’-TCCATCTCCGATTCAGTCCCT-3’
Hif1a (Forward)	5’-GAACGTCGAAAAGAAAAGTCTCG-3’

Hif1a (Reverse)	5’-CCTTATCAAGATGCGAACTCACA-3’
CD74 (Forward)	5’-GACGAGAACGGCAACTATCTG-3’
CD74 (Reverse)	5’-GTTGGGGAAGACACACCAGC-3’
Cox2 (Forward)	5’-ATGCTGACTATGGCTACAAAAGC-3’
Cox2 (Reverse)	5’-TCGGGCAATCATCAGGCAC-3’
MMP2 (Forward)	5’-CCCACTGCGGTTTTCTCGAAT-3’
MMP2 (Reverse)	5’-CAAAGGGGTATCCATCGCCAT-3’
c-Myc (Forward)	5’-GGCTCCTGGCAAAAGGTCA-3’
c-Myc (Reverse)	5’-CTGCGTAGTTGTGCTGATGT-3’
Tiam (Forward)	5’-CCTGTGTCTTACACTGACTCTTC-3’
Tiam (Reverse)	5’-CATCCCCGTAAAGCCTGCTC-3’
Kiaa (Forward)	5’-GAACGCCACTCAGCTTTTGC-3’
Kiaa (Reverse)	5’-GAAGCACTTATGTTGGGGTCTT-3’
Ccnd2 (Forward)	5’-TTTGCCATGTACCCACCGTC-3’
Ccnd2 (Reverse)	5’-AGGGCATCACAAGTGAGCG-3’
Sox4 (Forward)	5’-AGCGACAAGATCCCTTTCATTC-3’
Sox4 (Reverse)	5’-CGTTGCCGGACTTCACCTT-3’
Fn14 (Forward)	5’-CTGCTAACTGTTCCTCGGCT-3’
Fn14 (Reverse)	5’-GCCTTGGCAATCCTTTCACA-3’
Sox2 (Forward)	5’-GCCGAGTGGAAACTTTTGTCG-3’
Sox2 (Reverse)	5’-GGCAGCGTGTACTTATCCTTCT-3’
Hes6 (Forward)	5’-AGCAGGAGCCTGACTCAGTT-3’
Hes6 (Reverse)	5’-AGCTCCTGAACCATCTGCTC-3’
Hey1 (Forward)	5’-GTTCGGCTCTAGGTTCCATGT-3’
Hey1 (Reverse)	5’-CGTCGGCGCTTCTCAATTATTC-3’
Hes1 (Forward)	5’-TCAACACGACACCGGATAAAC-3’
Hes1 (Reverse)	5’-GCCGCGAGCTATCTTTCTTCA-3’
Gli1 (Forward)	5’-TGGATATGATGGTTGGCAAGTG-3’
Gli1 (Reverse)	5’-ACAGACTCAGGCTCAGGCTTCT-3’
Ptch1 (Forward)	5’-CCACAGAAGCGCTCCTACA-3’
Ptch1 (Reverse)	5’-CTGTAATTTCGCCCCTTCC-3’
Gli3 (Forward)	5’-GAAGTGCTCCACTCGAACAGA-3’
Gli3 (Reverse)	5’-GTGGCTGCATAGTGATTGCG-3’
JunB (Forward)	5’-ACAAACTCCTGAAACCGAGCC-3’
JunB (Reverse)	5’-CGAGCCCTGACCAGAAAAGTA-3’
Cbfa1 (Forward)	5’-TGGTTACTGTCATGGCGGGTA-3’
Cbfa1 (Reverse)	5’-TCTCAGATCGTTGAACCTTGCTA-3’
Msx2 (Forward)	5’-ATGGCTTCTCCGTCCAAAGG-3’
Msx2 (Reverse)	5’-CGGCTTCTTGTCGGACATGA-3’
Smad7 (Forward)	5’-TTCCTCCGCTGAAACAGGG-3’
Smad7 (Reverse)	5’-CCTCCCAGTATGCCACCAC-3’
Nanog (Forward)	5’-TTTGTGGGCCTGAAGAAAACT-3’
Nanog (Reverse)	5’-AGGGCTGTCCTGAATAAGCAG-3’
Runx1 (Forward)	5’-CTGCCCATCGCTTTCAAGGT-3’
Runx1 (Reverse)	5’-GCCGAGTAGTTTTCATCATTGCC-3’
-428 (Forward) (YAP1p)	5’-GCGAACTGGAAGCGCCTTTC-3’
-281 (Reverse) (YAP1p)	5’-GAAGCGTGCCCCGTATTCT-3’
-803 (Forward) (YAP1p)	5’-GTTGCGGCTTCCAGTGACTA-3’
-541 (Reverse) (YAP1p)	5’-AAGCCGCGAGGATAGATTGG-3’
-992 (Forward) (YAP1p)	5’-GGATGACCTCCTTGCCCATT-3’
-925 (Reverse) (YAP1p)	5’-CCTACCCCTGCTATCCCTGT-3’
-1334 (Forward) (YAP1p)	5’-ACCTTTTTGCTCACGGACTT-3’
-1240 (Reverse) (YAP1p)	5’-GCACAAGCCCATTCTAAGGAAAT-3’

-1486 (Forward) (YAP1p)	5'-GACAGGAAACTGGGGGTTCA-3'
-1436 (Reverse) (YAP1p)	5'-GCAGGCCTCAGACACTCAAA-3'
-1959 (Forward) (YAP1p)	5'-GCATAGTTTCTGCCCAAAGGTT-3'
-1900 (Reverse) (YAP1p)	5'-AGGGACCCTGTTGGTAGTCC-3'
-2578 (Forward) (YAP1p)	5'-ATCCCCTACTACACCGTGGTT-3'
-2341 (Reverse) (YAP1p)	5'-TGGGATTGGAATAGAAGCTGTCC-3'
-2970 (Forward) (YAP1p)	5'-GAAGGGAGTGATAGGTTGCAGA -3'
-2840 (Reverse) (YAP1p)	5'-TCGTCTGTACTCTGCATCATCC-3'
-3420 (Forward) (YAP1p)	5'-ACAATGAATTACGATTTCCA-3'
-3305 (Reverse) (YAP1p)	5'-GTGAGAAATAAAATTCTAG -3'
-3842 (Forward) (YAP1p)	5'-AGCTTAGGCGTTGGAATAAAACA -3'
-3776 (Reverse) (YAP1p)	5'-TCAATCCCGTTCTCTCATGGATT-3'
-4441 (Forward) (YAP1p)	5'-CACCATGTTGGCCAGGCTGGTG-3'
-4287 (Reverse) (YAP1p)	5'-GATGGAGCCATTGCACTCCAGC-3'
-4906 (Forward) (YAP1p)	5'-AAATGTGGAAGGACACTAATGC-3'
-4857 (Reverse) (YAP1p)	5'-TAACACCTTGGTTTCTGGGGC-3'
-2700 (Forward) (LncBRMp)	5'-GGGAATTGGTGTGGCAGGGGGA-3'
-2500 (Reverse) (LncBRMp)	5'-ATAGTCTACACAGGTCAGCCTC-3'

YAP1p: *YAP1* promoter

LncBRMp: *LncBRM* promoter.

Supplementary Table 4. shRNA sequences used in this study

shRNA	Sequence
shLncBRM#1	5'-GGAATATTGTCGACTTAAC-3'
shLncBRM#2	5'-GGACCACTAGGTTTCATAT-3'
sh16757601	5'-GGAATATGATGAATGATCA-3'
sh16934218	5'-GGAGGAAGTTGTTGGCTGT-3'
sh16894703	5'-GCACCAAGACTCCTGATCA-3'
sh17116795	5'-GGATGAATGAGTGCAGAGA-3'
sh17104376	5'-GCATCACCATAGCCATAAG-3'
sh17117451	5'-GCAGAAGAGTGAGGAGTTA-3'
sh16866275	5'-GCCTGAAGTCATAACCAGA-3'
sh16703431	5'-GGACCACACTGAACTCTTG-3'
sh16890400	5'-GGTTTATGATGCCAGCAGA-3'
shLncTcf7	5'-AGCCAACATTGTTGGTTAT-3'
